# 2-[(2,6-Dichloro­benz­yl)amino]-*N*-(4-methyl­thia­zol-2-yl)acetamide

**DOI:** 10.1107/S1600536810003089

**Published:** 2010-02-03

**Authors:** Jie Luo, Gui-Long Zhao, Hua Shao, Yu-Li Wang, Bao-Han Qu

**Affiliations:** a College of Chemistry and Pharmaceutical Sciences, Qingdao Agricultural University, Qingdao 266109, People’s Republic of China; b Tianjin Key Laboratory of Molecular Design and Drug Discovery, Tianjin Institute of Pharmaceutical Research, Tianjin 300193, People’s Republic of China

## Abstract

In the title compound, C_13_H_13_Cl_2_N_3_OS, the thia­zole and benzene rings are roughly parallel to one another in two layers [dihedral angle = 5.08 (2)°] because the N—C—C—N—C chain that links the two rings is folded [N—C—C—N torsion angle = 12.0 (2)°] rather than fully extended. An intra­molecular N—H⋯N inter­action occurs. In the crystal, weak inter­molecular N—H⋯N and C—H⋯O inter­actions are present and π–π inter­actions are indicated by the short distances [3.507 (3)–3.665 (2) Å] between the centroids of the thia­zole and benzene rings.

## Related literature

For details of the biological activity of Dipeptidyl peptidase IV (DPP-IV) inhibitors, see: Cheon *et al.* (2005 ▶); Kondo *et al.* (2007 ▶); Sakashita *et al.* (2006 ▶); Zhan *et al.* (2009 ▶). For bond-length data, see: Allen *et al.* (1987 ▶).
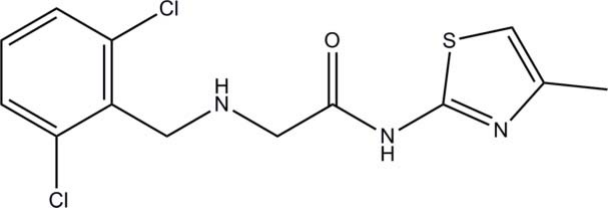


## Experimental

### 

#### Crystal data

C_13_H_13_Cl_2_N_3_OS
*M* *_r_* = 330.22
Monoclinic, 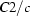

*a* = 14.008 (4) Å
*b* = 18.133 (5) Å
*c* = 11.390 (3) Å
β = 97.341 (3)°
*V* = 2869.4 (14) Å^3^
*Z* = 8
Mo *K*α radiation
μ = 0.60 mm^−1^
*T* = 113 K
0.22 × 0.18 × 0.16 mm

#### Data collection

Rigaku Saturn diffractometer
Absorption correction: multi-scan (*CrystalClear*; Rigaku, 2007 ▶) *T*_min_ = 0.880, *T*_max_ = 0.911
11103 measured reflections
3371 independent reflections
3150 reflections with *I* > 2σ(*I*)
*R*_int_ = 0.036

#### Refinement

*R*[*F* ^2^ > 2σ(*F* ^2^)] = 0.056
*wR*(*F* ^2^) = 0.131
*S* = 1.20
3371 reflections
191 parameters
H atoms treated by a mixture of independent and constrained refinement
Δρ_max_ = 0.65 e Å^−3^
Δρ_min_ = −0.83 e Å^−3^

### 

Data collection: *CrystalClear* (Rigaku, 2007 ▶); cell refinement: *CrystalClear*; data reduction: *CrystalClear*; program(s) used to solve structure: *SHELXS97* (Sheldrick, 2008 ▶); program(s) used to refine structure: *SHELXL97* (Sheldrick, 2008 ▶); molecular graphics: *SHELXTL* (Sheldrick, 2008 ▶); software used to prepare material for publication: *SHELXTL*.

## Supplementary Material

Crystal structure: contains datablocks I, global. DOI: 10.1107/S1600536810003089/fl2286sup1.cif

Structure factors: contains datablocks I. DOI: 10.1107/S1600536810003089/fl2286Isup2.hkl

Additional supplementary materials:  crystallographic information; 3D view; checkCIF report

## Figures and Tables

**Table 1:** Hydrogen-bond geometry (Å, °)

*D*—H⋯*A*	*D*—H	H⋯*A*	*D*⋯*A*	*D*—H⋯*A*
N3—H3⋯N1^i^	0.89 (2)	2.32 (2)	3.130 (2)	150.9 (17)
C7—H7*B*⋯O1^ii^	0.99	2.53	3.3233 (19)	137
N2—H2⋯N3	0.84 (2)	2.26 (2)	2.6742 (19)	111 (2)

Symmetry codes: (i) 
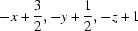
; (ii) 
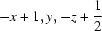
.

**Fig. 1.**
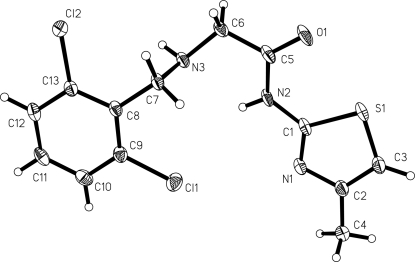
The molecular structure of (I), with atom labels and 40% probability displacement ellipsoids for non-H atoms.

**Fig. 2.**
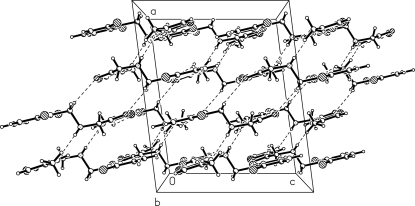
A packing diagram of the molecule of the title compound, view down b axis. Hydrogen bonds are shown as dashed lines.

## supplementary crystallographic information

### Comment

Dipeptidyl peptidase IV (DPP-IV) inhibitors are a new class of antidiabetic agents (Cheon *et al.*, 2005; Kondo *et al.*, 2007; Sakashita *et al.*, 2006) and the title compound was prepared as a novel DPP-IV inhibitor (Zhan *et al.*, 2009). We reported the crystal structure here.

In title compound, all bond lengths in the molecular are normal (Allen *et al.*, 1987). Atoms C4—C6/N2/N3/O1 lie in thiazole (C1—C3/N1/S1) plane with maximun least squares plane deviation for C4 0.065 (2) Å. Thiazole ring (C1—C3/N1/S1) and benzene ring (C8—C13) are essentially parallel to one another (dihedral angle of 5.08 (2) °) because the N—C—C—N—C chain that links the two rings is folded ((N—C—C—N torsion angle of 12.0 (2) °) rather than fully extended. π—π interactions are indicated by the short distance (Cg1···Cg1 distance of 3.665 (2) Å, symmetry code: 1-x,y,1/2-z; Cg1···Cg2 distance of 3.766 (3) Å, symmetry code: 1/2-x,1/2-y,-z; Cg1···Cg2 distance of 3.507 (3) Å, symmetry code: 1-x,y,-1/2-z) between the centroids of the thiazole rings C1—C3/N1/S1 (Cg1) and benzene rings C8—C13 (Cg2) (Table 1). There are intramolecular interaction and weaker N—H···N, C—H···O intermolecular interactions, which stabilized the structure (Table 1).

### Experimental

A round-bottomed flask was charged with [(2,6-dichlorophenyl)methyl]amine (1.76 g, 10 mmol), 2-[(2-chloroacetyl)amino]-4-methylthiazole (1.91 g, 10 mmol), triethylamine (1.21 g, 12 mmol) and THF (20 ml), and the resulting mixture was stirred at room temperature over night. The reaction mixture was concentrated on a rotary evaporator and diluted with 100 ml of dichloromethane, and the organic solution thus obtained was washed with saturated brine, dried over sodium sulfate and evaporated on a rotary evaporator to afford a solid residue, which was triturated with a mixed solvent consisting of 5 ml of dichloromethane and 10 ml of ethyl acetate and filtered. The crystals were collected and dried to yield the title compound as colorless crystals 2.51 g (Yield 76.1%). Crystals suitable for single-crystal X-ray diffraction were obtained via slow evaporation at room temperature of a solution of the pure title compound in dichloromethane.

### Refinement

All H atoms were found on difference maps. The H atoms of secondary amine were refined freely, giveing 0.84 or 0.89 Å, and C—H hyrdogens in the final cycles of refinement using a riding model, giveing 0.95-0.99 Å, with U_iso_(H) = 1.2U_eq_(C) and 1.5U_eq_(C) for the methyl H atoms.

### Crystal data

**Table:**

C_13_H_13_Cl_2_N_3_OS	*F*(000) = 1360
*M**_r_* = 330.22	*D*_x_ = 1.529 Mg m^−^^3^
Monoclinic, *C*2/*c*	Mo *K*α radiation, λ = 0.71075 Å
Hall symbol: -C 2yc	Cell parameters from 5153 reflections
*a* = 14.008 (4) Å	θ = 1.8–27.9°
*b* = 18.133 (5) Å	µ = 0.60 mm^−^^1^
*c* = 11.390 (3) Å	*T* = 113 K
β = 97.341 (3)°	Prism, colorless
*V* = 2869.4 (14) Å^3^	0.22 × 0.18 × 0.16 mm
*Z* = 8	

### Data collection

**Table:**

Rigaku Saturn diffractometer	3371 independent reflections
Radiation source: rotating anode	3150 reflections with *I* > 2σ(*I*)
multilayer	*R*_int_ = 0.036
Detector resolution: 14.63 pixels mm^-1^	θ_max_ = 27.9°, θ_min_ = 1.9°
ω and φ scans	*h* = −18→18
Absorption correction: multi-scan (*CrystalClear*; Rigaku, 2007)	*k* = −23→18
*T*_min_ = 0.880, *T*_max_ = 0.911	*l* = −13→14
11103 measured reflections	

### Refinement

**Table:**

Refinement on *F*^2^	Secondary atom site location: difference Fourier map
Least-squares matrix: full	Hydrogen site location: inferred from neighbouring sites
*R*[*F*^2^ > 2σ(*F*^2^)] = 0.056	H atoms treated by a mixture of independent and constrained refinement
*wR*(*F*^2^) = 0.131	*w* = 1/[σ^2^(*F*_o_^2^) + (0.0837*P*)^2^ + 0.6909*P*] where *P* = (*F*_o_^2^ + 2*F*_c_^2^)/3
*S* = 1.20	(Δ/σ)_max_ < 0.001
3371 reflections	Δρ_max_ = 0.65 e Å^−^^3^
191 parameters	Δρ_min_ = −0.83 e Å^−^^3^
0 restraints	Extinction correction: *SHELXL97* (Sheldrick, 2008), Fc^*^=kFc[1+0.001xFc^2^λ^3^/sin(2θ)]^-1/4^
Primary atom site location: structure-invariant direct methods	Extinction coefficient: 0.086 (3)

### Special details

**Table:**

Geometry. All esds (except the esd in the dihedral angle between two l.s. planes) are estimated using the full covariance matrix. The cell esds are taken into account individually in the estimation of esds in distances, angles and torsion angles; correlations between esds in cell parameters are only used when they are defined by crystal symmetry. An approximate (isotropic) treatment of cell esds is used for estimating esds involving l.s. planes.
Refinement. Refinement of *F*^2^ against ALL reflections. The weighted *R*-factor wR and goodness of fit *S* are based on *F*^2^, conventional *R*-factors *R* are based on *F*, with *F* set to zero for negative *F*^2^. The threshold expression of *F*^2^ > σ(*F*^2^) is used only for calculating *R*-factors(gt) etc. and is not relevant to the choice of reflections for refinement. *R*-factors based on *F*^2^ are statistically about twice as large as those based on *F*, and *R*- factors based on ALL data will be even larger.

### Fractional atomic coordinates and isotropic or equivalent isotropic displacement parameters (Å^2^)

**Table:**

	*x*	*y*	*z*	*U*_iso_*/*U*_eq_	
Cl1	0.59889 (3)	0.30694 (2)	0.64087 (4)	0.03507 (18)	
Cl2	0.60026 (3)	0.00844 (2)	0.65202 (3)	0.03000 (17)	
S1	0.60179 (3)	0.37651 (2)	0.15270 (3)	0.02879 (17)	
O1	0.59946 (9)	0.22476 (8)	0.16770 (10)	0.0371 (3)	
N2	0.65223 (10)	0.28348 (8)	0.33975 (11)	0.0262 (3)	
N1	0.66017 (10)	0.41118 (7)	0.37000 (11)	0.0260 (3)	
N3	0.66669 (9)	0.15932 (7)	0.46748 (11)	0.0244 (3)	
C1	0.64101 (10)	0.35505 (9)	0.29875 (12)	0.0242 (3)	
C2	0.64316 (12)	0.47666 (9)	0.30816 (14)	0.0284 (3)	
C3	0.61282 (13)	0.46827 (10)	0.19104 (15)	0.0325 (4)	
H3A	0.5994	0.5080	0.1370	0.039*	
C4	0.65614 (15)	0.54732 (10)	0.37486 (15)	0.0368 (4)	
H4A	0.6702	0.5869	0.3211	0.055*	
H4B	0.7097	0.5425	0.4387	0.055*	
H4C	0.5971	0.5590	0.4087	0.055*	
C5	0.63006 (11)	0.22221 (9)	0.27318 (13)	0.0282 (4)	
C6	0.64796 (12)	0.15012 (9)	0.33971 (13)	0.0290 (4)	
H6A	0.5911	0.1178	0.3207	0.035*	
H6B	0.7037	0.1250	0.3119	0.035*	
C7	0.57765 (11)	0.15610 (8)	0.52297 (13)	0.0239 (3)	
H7A	0.5421	0.1103	0.4986	0.029*	
H7B	0.5360	0.1986	0.4964	0.029*	
C8	0.60135 (10)	0.15779 (8)	0.65549 (12)	0.0216 (3)	
C9	0.61402 (11)	0.22376 (8)	0.71857 (13)	0.0251 (3)	
C10	0.63758 (11)	0.22652 (10)	0.84107 (14)	0.0299 (4)	
H10	0.6454	0.2726	0.8809	0.036*	
C11	0.64947 (12)	0.16110 (10)	0.90375 (14)	0.0299 (4)	
H11	0.6655	0.1622	0.9873	0.036*	
C12	0.63813 (11)	0.09389 (9)	0.84525 (14)	0.0275 (3)	
H12	0.6462	0.0488	0.8880	0.033*	
C13	0.61481 (10)	0.09381 (8)	0.72306 (13)	0.0232 (3)	
H2	0.6752 (16)	0.2782 (14)	0.411 (2)	0.049 (6)*	
H3	0.7065 (14)	0.1245 (11)	0.5004 (18)	0.030 (5)*	

### Atomic displacement parameters (Å^2^)

**Table:**

	*U*^11^	*U*^22^	*U*^33^	*U*^12^	*U*^13^	*U*^23^
Cl1	0.0473 (3)	0.0242 (2)	0.0324 (3)	−0.00894 (14)	−0.0001 (2)	0.00170 (13)
Cl2	0.0396 (3)	0.0237 (3)	0.0275 (3)	−0.00282 (13)	0.00714 (17)	0.00110 (12)
S1	0.0322 (3)	0.0420 (3)	0.0119 (2)	−0.00337 (14)	0.00174 (16)	0.00325 (13)
O1	0.0493 (8)	0.0486 (8)	0.0121 (5)	−0.0193 (5)	−0.0016 (5)	−0.0025 (5)
N2	0.0355 (7)	0.0315 (7)	0.0105 (6)	−0.0120 (5)	−0.0011 (5)	0.0016 (5)
N1	0.0302 (7)	0.0323 (7)	0.0157 (6)	−0.0046 (5)	0.0031 (5)	0.0022 (5)
N3	0.0305 (7)	0.0278 (7)	0.0147 (6)	−0.0082 (5)	0.0028 (5)	−0.0007 (4)
C1	0.0244 (7)	0.0353 (8)	0.0131 (7)	−0.0071 (6)	0.0029 (5)	0.0024 (5)
C2	0.0309 (8)	0.0345 (8)	0.0211 (8)	0.0034 (6)	0.0079 (6)	0.0043 (6)
C3	0.0397 (9)	0.0384 (9)	0.0200 (8)	0.0065 (7)	0.0066 (6)	0.0064 (6)
C4	0.0546 (11)	0.0321 (8)	0.0253 (8)	0.0080 (7)	0.0110 (7)	0.0037 (6)
C5	0.0331 (8)	0.0380 (8)	0.0134 (7)	−0.0158 (6)	0.0027 (6)	−0.0025 (6)
C6	0.0411 (9)	0.0298 (8)	0.0161 (8)	−0.0137 (6)	0.0033 (6)	−0.0048 (6)
C7	0.0282 (7)	0.0285 (8)	0.0146 (7)	−0.0088 (5)	0.0007 (5)	0.0015 (5)
C8	0.0222 (7)	0.0265 (8)	0.0160 (7)	−0.0061 (5)	0.0016 (5)	0.0002 (5)
C9	0.0271 (7)	0.0277 (7)	0.0201 (7)	−0.0056 (5)	0.0008 (6)	0.0005 (5)
C10	0.0297 (8)	0.0367 (8)	0.0226 (8)	−0.0045 (6)	0.0007 (6)	−0.0084 (6)
C11	0.0273 (8)	0.0471 (10)	0.0147 (7)	−0.0007 (6)	0.0007 (6)	−0.0007 (6)
C12	0.0262 (7)	0.0378 (9)	0.0190 (7)	0.0009 (6)	0.0042 (6)	0.0064 (6)
C13	0.0236 (7)	0.0272 (7)	0.0192 (7)	−0.0035 (5)	0.0044 (5)	0.0009 (5)

### Geometric parameters (Å, °)

**Table:**

Cl1—C9	1.7480 (16)	C4—H4B	0.9800
Cl2—C13	1.7465 (16)	C4—H4C	0.9800
S1—C3	1.7222 (19)	C5—C6	1.516 (2)
S1—C1	1.7282 (15)	C6—H6A	0.9900
O1—C5	1.2238 (19)	C6—H6B	0.9900
N2—C5	1.359 (2)	C7—C8	1.5038 (19)
N2—C1	1.381 (2)	C7—H7A	0.9900
N2—H2	0.84 (2)	C7—H7B	0.9900
N1—C1	1.308 (2)	C8—C13	1.392 (2)
N1—C2	1.385 (2)	C8—C9	1.395 (2)
N3—C6	1.4550 (19)	C9—C10	1.393 (2)
N3—C7	1.470 (2)	C10—C11	1.384 (3)
N3—H3	0.89 (2)	C10—H10	0.9500
C2—C3	1.356 (2)	C11—C12	1.388 (2)
C2—C4	1.489 (3)	C11—H11	0.9500
C3—H3A	0.9500	C12—C13	1.388 (2)
C4—H4A	0.9800	C12—H12	0.9500
			
C3—S1—C1	88.07 (8)	C5—C6—H6A	108.9
C5—N2—C1	124.86 (13)	N3—C6—H6B	108.9
C5—N2—H2	118.5 (17)	C5—C6—H6B	108.9
C1—N2—H2	116.6 (17)	H6A—C6—H6B	107.7
C1—N1—C2	110.07 (13)	N3—C7—C8	109.89 (11)
C6—N3—C7	111.78 (12)	N3—C7—H7A	109.7
C6—N3—H3	111.3 (13)	C8—C7—H7A	109.7
C7—N3—H3	108.1 (13)	N3—C7—H7B	109.7
N1—C1—N2	121.05 (13)	C8—C7—H7B	109.7
N1—C1—S1	115.90 (12)	H7A—C7—H7B	108.2
N2—C1—S1	123.05 (11)	C13—C8—C9	115.51 (13)
C3—C2—N1	114.58 (15)	C13—C8—C7	122.35 (13)
C3—C2—C4	126.94 (15)	C9—C8—C7	122.11 (13)
N1—C2—C4	118.44 (14)	C10—C9—C8	123.02 (14)
C2—C3—S1	111.37 (12)	C10—C9—Cl1	118.29 (12)
C2—C3—H3A	124.3	C8—C9—Cl1	118.69 (11)
S1—C3—H3A	124.3	C11—C10—C9	118.91 (15)
C2—C4—H4A	109.5	C11—C10—H10	120.5
C2—C4—H4B	109.5	C9—C10—H10	120.5
H4A—C4—H4B	109.5	C10—C11—C12	120.41 (15)
C2—C4—H4C	109.5	C10—C11—H11	119.8
H4A—C4—H4C	109.5	C12—C11—H11	119.8
H4B—C4—H4C	109.5	C11—C12—C13	118.67 (14)
O1—C5—N2	122.92 (15)	C11—C12—H12	120.7
O1—C5—C6	122.55 (14)	C13—C12—H12	120.7
N2—C5—C6	114.51 (13)	C12—C13—C8	123.46 (14)
N3—C6—C5	113.47 (13)	C12—C13—Cl2	117.64 (12)
N3—C6—H6A	108.9	C8—C13—Cl2	118.89 (11)
			
C2—N1—C1—N2	−179.90 (14)	N3—C7—C8—C13	−91.91 (15)
C2—N1—C1—S1	0.25 (17)	N3—C7—C8—C9	86.22 (17)
C5—N2—C1—N1	176.97 (15)	C13—C8—C9—C10	−0.6 (2)
C5—N2—C1—S1	−3.2 (2)	C7—C8—C9—C10	−178.88 (14)
C3—S1—C1—N1	0.33 (13)	C13—C8—C9—Cl1	179.82 (11)
C3—S1—C1—N2	−179.52 (14)	C7—C8—C9—Cl1	1.57 (19)
C1—N1—C2—C3	−0.9 (2)	C8—C9—C10—C11	0.3 (2)
C1—N1—C2—C4	177.06 (14)	Cl1—C9—C10—C11	179.80 (12)
N1—C2—C3—S1	1.16 (19)	C9—C10—C11—C12	0.1 (2)
C4—C2—C3—S1	−176.61 (15)	C10—C11—C12—C13	0.1 (2)
C1—S1—C3—C2	−0.81 (13)	C11—C12—C13—C8	−0.5 (2)
C1—N2—C5—O1	1.8 (3)	C11—C12—C13—Cl2	−179.61 (12)
C1—N2—C5—C6	−179.27 (14)	C9—C8—C13—C12	0.8 (2)
C7—N3—C6—C5	90.42 (15)	C7—C8—C13—C12	179.01 (14)
O1—C5—C6—N3	−169.05 (15)	C9—C8—C13—Cl2	179.86 (11)
N2—C5—C6—N3	12.0 (2)	C7—C8—C13—Cl2	−1.90 (19)
C6—N3—C7—C8	174.04 (12)		

### Hydrogen-bond geometry (Å, °)

**Table:**

*D*—H···*A*	*D*—H	H···*A*	*D*···*A*	*D*—H···*A*
N3—H3···N1^i^	0.89 (2)	2.32 (2)	3.130 (2)	150.9 (17)
C7—H7B···O1^ii^	0.99	2.53	3.3233 (19)	137
N2—H2···N3	0.84 (2)	2.26 (2)	2.6742 (19)	111 (2)
Cg1—···.Cg1^ii^	.	.	3.665 (2)	.
Cg1—···.Cg2^iii^	.	.	3.766 (3)	.
Cg2—···.Cg2^iv^	.	.	3.507 (3)	.

Symmetry codes: (i) −*x*+3/2, −*y*+1/2, −*z*+1; (ii) −*x*+1, *y*, −*z*+1/2; (iii) −*x*+1/2, −*y*+1/2, −*z*; (iv) −*x*+1, *y*, −*z*−1/2.
